# Uncovering potential targets for antibody-drug conjugates in the treatment of gynecologic malignancies

**DOI:** 10.3389/fphar.2025.1525733

**Published:** 2025-02-19

**Authors:** Yuying Jiang, Yuance Xu, Junqi He, Lei Sui, Tian Li, Nannan Xia, Qin Yao

**Affiliations:** ^1^ Department of Obstetrics and Gynecology, The Affiliated Hospital of Qingdao University, Qingdao, China; ^2^ Department of Obstetrics and Gynecology, Qingdao Central Hospital, University of Health and Rehabilitation Sciences, Qingdao, China

**Keywords:** antibody-drug conjugates (ADCs), target prediction, targeted therapy, gynecological malignancies, new therapies

## Abstract

**Background:**

Antibody-drug conjugates (ADCs) play an important role in the targeted therapy of gynecological malignancies. The purpose of this study was to investigate the expression of targets in gynecologic malignancies in order to predict the selection of targets for the development of antibody-drug conjugates.

**Methods:**

In this article, we identified existing ADCs and their targets through clinical trial databases and public genomic datasets, performed differential analysis of tumor antigen targets (TATs) expression between tumor and normal tissues, and evaluated the necessity of the targets for tumor cell lines.

**Results:**

In gynecologic malignancies, we have identified several highly expressed TATs, some of which have been targeted by FDA-approved ADCs, such as TROP2 and Nectin-4, although these drugs have not been approved for the treatment of gynecologic cancers. At the same time, we also observed that some targets of ADCs that have not yet been approved by the FDA also show high expression levels in gynecologic malignancies tissues, such as MSLN, ERBB3, NaPi2b, etc. Furthermore, we identified TATs with high expression levels in various pathological subtypes of ovarian, endometrial, and cervical cancer. Notably, some TATs are crucial to the survival of tumor cells, such as CD71, TOP1, and TDGF1, which are essential for the survival of ovarian, endometrial, cervical, and other tumor cells.

**Conclusion:**

We have innovatively predicted the potential targets of ADCs in treating gynecological malignancies and provided a new perspective on applying some FDA-approved ADCs in indications for gynecological cancers.

## 1 Introduction

Gynecological cancers, including ovarian cancer, endometrial cancer, cervical cancer, etc., pose a significant threat to women’s health. At present, the treatment methods for gynecological malignancies mainly include surgery, chemotherapy, and targeted drug therapy. However, many patients may develop drug resistance after receiving chemotherapy, which limits the long-term efficacy of chemotherapy. In addition, the widespread cytotoxicity of traditional chemotherapy drugs not only eliminates cancer cells but also affects normal cells, causing a series of side effects. Although targeted therapy is more precise, its effectiveness is often limited to specific types of tumors and may face adverse pharmacokinetics and drug resistance issues in long-term use.

We aim to improve the cure rate, prolong patient survival, and enhance patients’ overall quality of life. In the face of these problems and challenges, we need to develop new targeted therapeutic drugs with higher targeting and fewer side effects and find innovative treatment strategies to reduce the recurrence risk. In 1907, German Nobel laureate Paul Ehrlich proposed a compound that could selectively target pathogenic microorganisms such as bacteria while protecting normal cells, a concept known as the “magic bullet” (Strebhardt and Ullrich, 2008). According to his hypothesis, an innovative treatment method emerged in the field of oncology, namely, antibody-drug conjugates (ADCs), which were first used to treat advanced cancer patients many years ago (Ford et al., 1983). This drug, which has a different mechanism of action from conventional tumor chemotherapy, has attracted much attention in the industry. Multiple clinical trial results have shown that ADCs have the advantages of high targeting, high specificity, and activity compared with standard treatment and are known as “biological missile” (Veneziani et al., 2024).

ADCs consist of three key components: antibodies, chemical linkers, and cytotoxic payloads (Phuna et al., 2024). The reason why ADC is described as a “biological missile” for cancer treatment is that when antibodies specifically bind to antigens on the surface of target cells, ADC is internalized, releasing payloads and exerting cytotoxicity. The ability to selectively deliver cytotoxic drugs to tumor cells and eliminate them without producing severe off-target toxic effects, with fewer side effects (Drago et al., 2021; Riccardi et al., 2023). The successful development of ADCs depends on the correct combination of three components, including designing monoclonal antibodies targeting specific tumor targets, using appropriate linkers, and selecting appropriate payloads (Dumontet et al., 2023). Using monoclonal antibodies to guide the release of active but toxic compounds is beneficial for patients (Nieto-Jiménez et al., 2023). In this article, we mainly explore the selection of monoclonal antibodies. Regarding the selection of specific monoclonal antibodies, in addition to the particular design or structure of the antibody itself, the ability of the antibody Fab region to target antigens expressed on tumor cells is the main determinant of anti-tumor activity and selectivity (Chari, 2008). In this case, the best option will be the use of an antibody against a tumor antigen targets (TATs) only expressed in the tumor, thereby exhibiting better anti-tumor activity (Boghaert et al., 2022).

Approved ADCs have shown significant clinical efficacy in increasing time event endpoints, such as progression-free survival (PFS) or overall survival (OS). Especially for indications with significant TATs, such as Trastuzumab Emtansine and Trastuzumab Deruxtecan targeting ERBB2, the prognosis and quality of life of ERBB2-positive breast cancer patients have been improved (Díaz-Rodríguez et al., 2021). As of April 2024, 15 ADCs have been approved by the Food and Drug Administration (FDA) worldwide (Table 1), and there are still a large number of ADCs and combination chemotherapy, immunotherapy, and anti-angiogenic drugs undergoing clinical or preclinical research. ADCs, as a new class of anti-cancer drugs, have shown great potential in the treatment of advanced and recurrent gynecological malignancies. Currently, the FDA-approved ADCs used in the field of gynecological cancers are Tisotumab Vedotin (TV) for the treatment of cervical cancer and Mirvetuximab Soravtansine (MIRV) for the treatment of ovarian cancer (Zhang et al., 2024). These drugs are expected to become an essential means of improving the prognosis of patients with recurrent and advanced gynecological malignancies. Although these two drugs have shown potential in the treatment of gynecological cancers, in order to improve treatment efficacy and meet the treatment needs of more patients, we still need to explore and develop more new ADCs to broaden the options for gynecological cancer treatment.

**TABLE 1:** Information on 15 ADC drugs approved by the Food and Drug Administration (FDA) as of April 2024.

Drug	Target	Cytotoxic drug	Chemical linker	Indication	Date of first approved
Gemtuzumab Ozogamicin	CD33	Calicheamicin	Acid-unstable hydrazone tendon	CD33-positive Acute Myeloid Leukemia (AML)	2000.05
Brentuximab Vedotin	CD30	MMAE	Protease-cleavable linker	Hodgkin Lymphoma (HL)	2011.08
Trastuzumab Emtansine	ERBB2	DM1	Non-cleavable sulfide bond	ERBB2-positive Metastatic Breast Cancer	2013.02
Inotuzumab Ozogamicin	CD22	Calicheamicin	Acid-unstable hydrazone tendon	Relapsed/Refractory (R/R) B-cell Acute Lymphoblastic Leukemia (B-ALL)	2017.06
Moxetumomab Pasudotox	CD22	Pasudotox	Protease-cleavable linker	Hairy Cell Leukemia (HCL)	2018.09
Polatuzumab Vedotin	CD79b	MMAE	Protease-cleavable linker	Diffuse Large B-cell Lymphoma (DLBL)	2019.06
Enfortumab Vedotin	Nectin-4	MMAE	Protease-cleavable linker	Advanced Urothelial Carcinoma	2019.12
Trastuzumab Deruxtecan	ERBB2	Dxd	Protease-cleavable linker	ERBB2-positive Metastatic Breast Cancer	2019.12
Sacituzumab Govitecan	TROP2	SN-38	Acid-unstable carbonate bond	Triple Negative Breast Cancer (TNBC)	2020.04
Belantamab Mafodotin	BCMA	MMAF	Non-cleavable sulfide bond	Relapsed/Refractory Multiple Myeloma (RRMM)	2020.08
ASP-1929	EGFR	IRDye700DX	N/A	Recurrent/Metastatic (R/M) Head and Neck Squamous Cell Carcinoma (HNSCC)	2020.09
Loncastuximab Tesirine	CD19	PBD	Protease-cleavable linker	Relapsed/Refractory (R/R) Large B-cell Lymphoma (LBCL)	2021.04
Disitamab Vedotin	ERBB2	MMAE	Protease-cleavable linker	ERBB2-positive Gastric Cancer	2021.06
Tisotumab Vedotin-tftv	TF	MMAE	Protease-cleavable linker	Recurrent/Metastatic Cervical Cancer	2021.09
Mirvetuximab Soravtansine	FOLR1	DM4	Non-cleavable sulfide bond	FOLR1-positive, Platinum-resistant Epithelial Ovarian, Fallopian Tube, or Primary Peritoneal Cancer	2022.11

In this article, we evaluate the current clinical development of ADCs, with a particular focus on TATs that are highly expressed in gynecologic malignancies and common essential genes to inform the development of new target selection for ADCs.

## 2 Materials and methods

### 2.1 Identification and classification of ADC

By using ClinicalTrials.gov (clinicaltrials.gov), We searched for clinical studies that included both private and public funding. We specified the term “cancer” in the search field “diseases” and “antibody-drug conjugates” in “other terms”. We found 740 clinical trials in this study (last accessed June 2024, Supplementary File 1) and removed 75 clinical trials that included compounds used to detect and track cancer, as well as some clinical vaccine trials. In total, 665 clinical trials were included in the analysis. We analyzed 665 retrieved trials and found 275 ADCs.

In addition, by using the ADCdb (ADCdb: the database of antibody-drug conjugates (idrblab.net), last accessed August 2024) to search for these 275 ADC drugs again, we found the antibody names and antigen targets corresponding to these ADCs. We obtained a total of 89 antigen target information.

### 2.2 Evaluate the expression and dependence of ADC targets

Cancer Today (Cancer Today (iarc.fr), https://gco.iarc.fr/today/home, last accessed August 2024), an information platform of the International Agency for Research on Cancer (IARC), by visiting the website, we successfully identified the four most common types of current gynecological malignancies, including ovarian cancer, cervical cancer, endometrial cancer, and uterine sarcoma.

Gene Expression Profiling Interactive Analysis 2 (GEPIA 2 (cancer-pku.cn), last accessed in August 2024) is a gene expression analysis platform based on tumor and normal samples in TCGA and GTEx databases. We analyzed the expression of these targets in common gynecological cancers (cervical cancer, ovarian cancer, endometrial cancer, uterine sarcoma) tissues and normal tissues. We obtained expression data TPM (transcripts per million, TPM) for both tumor and normal tissues. TPM, as a standardized unit for measuring target gene expression, is a widely used method for quantifying gene expression levels (Li et al., 2019).

To investigate the expression of cancer subtypes, we also accessed the public dataset GENT2 database (GENT2 (appex.kr), http://gent2.appex.kr/gent2/, last accessed August 2024). This search platform can compile gene expression patterns of normal and tumor tissues in public gene expression datasets. It can be used to study differential expression and prognostic significance based on tumor subtypes. Data obtained were normalized by MAS5 and represented by log two of the Fold Change to simplify the representation (Park et al., 2019).

Universal Cancer Database (UALCAN (uab.edu), last accessed August 2024) is also a bioinformatics database that integrates transcriptome data from multiple public databases, such as TCGA and other high-throughput sequencing data, covering a variety of cancer types and samples. It provides gene expression data for multiple types of cancer. This database may provide rich data visualization options, such as box plots, which can intuitively analyze differential expressions.

DepMap (DepMap: The Cancer Dependency Map Project at Broad Institute, last accessed September 2024), which enables large-scale genetic screening using CRISPR-Cas9 and RNAi, can help researchers understand the effect of specific genes on specific cancer cell lines. We utilized DepMap to study the effect of a target on tumor cell viability to identify essential genes and strongly selective genes required for cancer cell survival (Tsherniak et al., 2017). From the scores provided by DepMap (Chronos for CRISPR and DEMETER2 for RNAi), we selected an average score of −0.4 as a threshold to screen out targets critical for tumor cell survival. This will lead to the development of anti-cancer drugs and personalized treatment strategies.

## 3 Results

### 3.1 A snapshot of the current clinical development stage of ADCs

**FIGURE 1:**
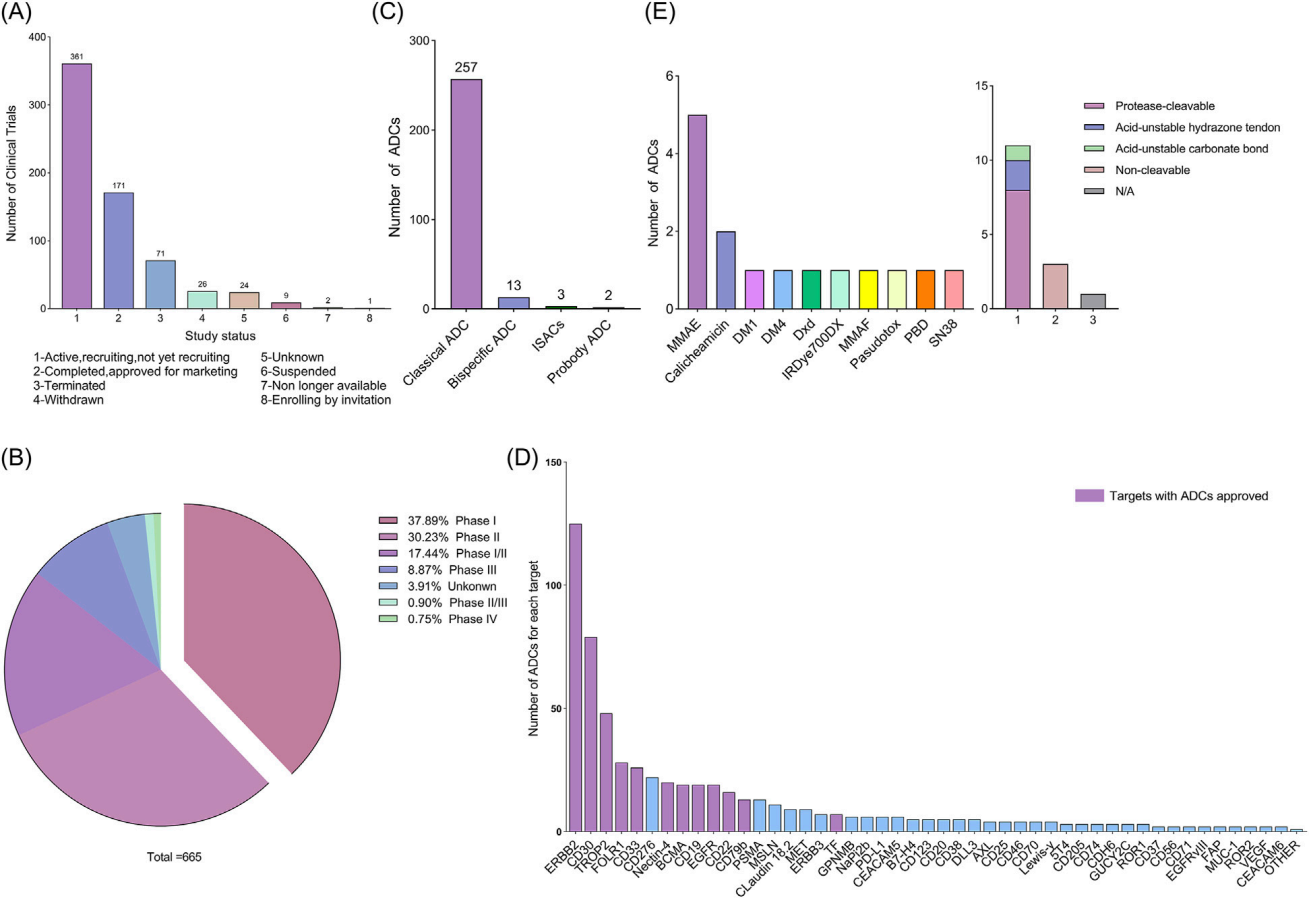
**(A)** Status of current clinical trials on antibody-drug conjugates (ADCs). **(B)** Classification of ADCs according to the phase of the clinical trials. **(C)** Classification of all ADCs in clinical trials according to their type. **(D)** Number of ADCs in a clinical trial for each target. Other: 4-1BB, ADAM9, ALCAM, CCR7, CD117, CD134, CD138, CD228, CD34, CD44, CD48, Claudin-6, DLK-1, DPEP3, EFNA4, EGF, ENPP3, EPHA5, FGFR2, FGFR3, FLT3, FN1, GPA33, GPR20, HAVCR1, IGF-1R, ITGB6, KAAG1, LIV-1, LRRC15, LYPD3, MUC-2, PTK7, SLAMF7, SLC44A4, SLITRK6, TDGF1, TM4SF1, TRPV6, FcRH5, TOP1 and A2aR. **(E)** Linkers and payloads of ADCs FDA-approved.

According to our research criteria, as described in the materials and methods, we identified 740 clinical trials related to ADCs. After screening, 665 clinical trials met the criteria and were included in the analysis (Supplementary File 2). We divided them into the following subgroups based on their research status (Figure 1A): 361 clinical trials are active trials, including those that have not yet been recruited and are currently being recruited; 171 clinical trials are in completion status or approved for marketing; 71 studies were terminated due to safety risks, lack of efficacy of experimental drugs, ethical issues, regulatory issues, changes in market demand, and other reasons; 26 projects were withdrawn; nine studies have been postponed; two studies are no longer available; one study requires an invitation to register. Additionally, it should be noted that 24 studies have a status of “unknown”. These data provide an overview of the current status of clinical trials for ADCs, reflecting the challenges and dynamic changes in the development process of ADCs.

In addition, clinical trials of ADCs are divided into phases I, II, III, and IV. Phase I and II clinical trials: These two phases are referred to as the early stages, during which researchers primarily evaluate the safety, tolerability, and optimal dosage of ADCs. Phase III clinical trials highlight new drug development and the stage of confirming therapeutic effects. Successful Phase III trials can support regulatory approval of drugs. Phase IV clinical trial: This phase monitors the drug’s long-term side effects and efficacy after being approved and widely used to evaluate its performance in clinical practice. Some ADCs have obtained regulatory approval for specific indications, while others are still developing. As of 12 June 2024, 85.56% (569/665) of clinical trials were in the early stages (Figure 1B), and 10.52% (70/665) were ongoing or had reached the Phase III. This data indicates that ADCs are still in the early development phase of clinical research.

Based on these studies as the primary source, we explored the structure of 275 ADCs involved in 665 clinical trials (Figure 1C). Of these, 257 ADCs had a traditional structure (monoclonal antibody-linker-cytotoxic payload). Only a few ADCs contain bispecific antibodies or specific immunostimulant. Bispecific antibodies (BsAb) typically refer to antibodies with two binding sites directed at two different antigens or epitopes. Specific immunostimulants can activate the innate immune system, promoting the initiation and recruitment of tumor-specific cells (Fu et al., 2024). At present, 13 bispecific ADCs have been developed, including M1231 designed for EGFR/MUC1, Glofitamab targeting both CD3 and CD20, Blinatumomab targeting CD3 and CD19, CD30 biAb-AATC targeting CD30 and CD3, etc.; ADCs containing specific immunostimulants are only three in number, and these ADCs are also known as immunostimulatory antibody conjugates (ISACs), including BDC-1001, SBT6050, and NJH395. These innovative ADCs demonstrate the potential for treating cancer by precisely targeting tumor cells. In addition, there are two types of probody ADCs, CX-2029 and CX-2009. They are designed to remain inactive until proteolytically activated in the tumor microenvironment, allowing ADCs to exhibit no toxicity when administered systemically, and when antibody in ADC bind to target cells expressing tumor antigens, the entire ADC is internalized by tumor cells, followed by the release of small molecule cytotoxins inside the cells, killing tumor cells in an efficient and active form. Although new ADCs have shown potential in cancer treatment, they are still in their infancy and face design and manufacturing complexities, as well as potential safety and efficacy issues that require further research and development.

By analyzing the 275 ADCs included in the study, we identified their targets and found 89 targets (Figure 1D). The most common targets of FDA approved ADC drugs include CD22, ERBB2, CD33, CD30, CD79b, Nectin-4, TROP2, CD19, Tissue Factor (TF), and FOLR1 (FRα). In addition, the cytotoxic drug for most ADCs is Monomethyl Auristatin E (MMAE, 33.3%), followed by Calicheamicin (13.3%). Finally, most of the ADCs had a cleavable linker (73.3%) compared with those wearing a non-cleavable one (20%). Among the cleavable ones, those with a protease cleavable linker were the most frequent (72.7%) (Figure 1E).

### 3.2 Specific TATs in gynecological cancers

At present, popular targets include CD family (CD33, CD30, CD22, CD79b, CD19), BCMA, ERBB2, TROP2, TF, Nectin-4, FOLR1, EGFR, PSMA, etc. So far, FDA-approved ADC targets for treating solid tumors include ERBB2, TROP2, FOLR1, Nectin-4, EGFR, and TF. Among them, ERBB2 is the most popular target for ADCs research.

Currently, ADCs are not widely used in the treatment of gynecological cancers. The FDA has approved two ADCs for gynecological cancers: Tisotumab Vedotin (TV) and Mirvetuximab Soravtansine (MIRV) (Zhang et al., 2024). TV is a TF-targeting ADC, commonly used for the treatment of recurrent or metastatic cervical cancer (Heitz et al., 2023); Mirvetuximab Soravtansine (MIRV) is a FOLR1-targeting ADC, which is used for the treatment of ovarian cancer, especially in patients with high expression of folate receptor alpha (FOLR1) who are resistant to platinum-based drugs (Moore et al., 2023). Trastuzumab Deruxtecan (T-DXd) is an ADC for targeting ERBB2. The FDA has accelerated approval of T-DXd for unresectable or metastatic ERBB2-positive solid tumors that have been systematically treated and for which no other treatment options exist. It has shown activity in ERBB2-positive breast cancer and is exploring its application in endometrial cancer (Meric-Bernstam et al., 2024).

**FIGURE 2:**
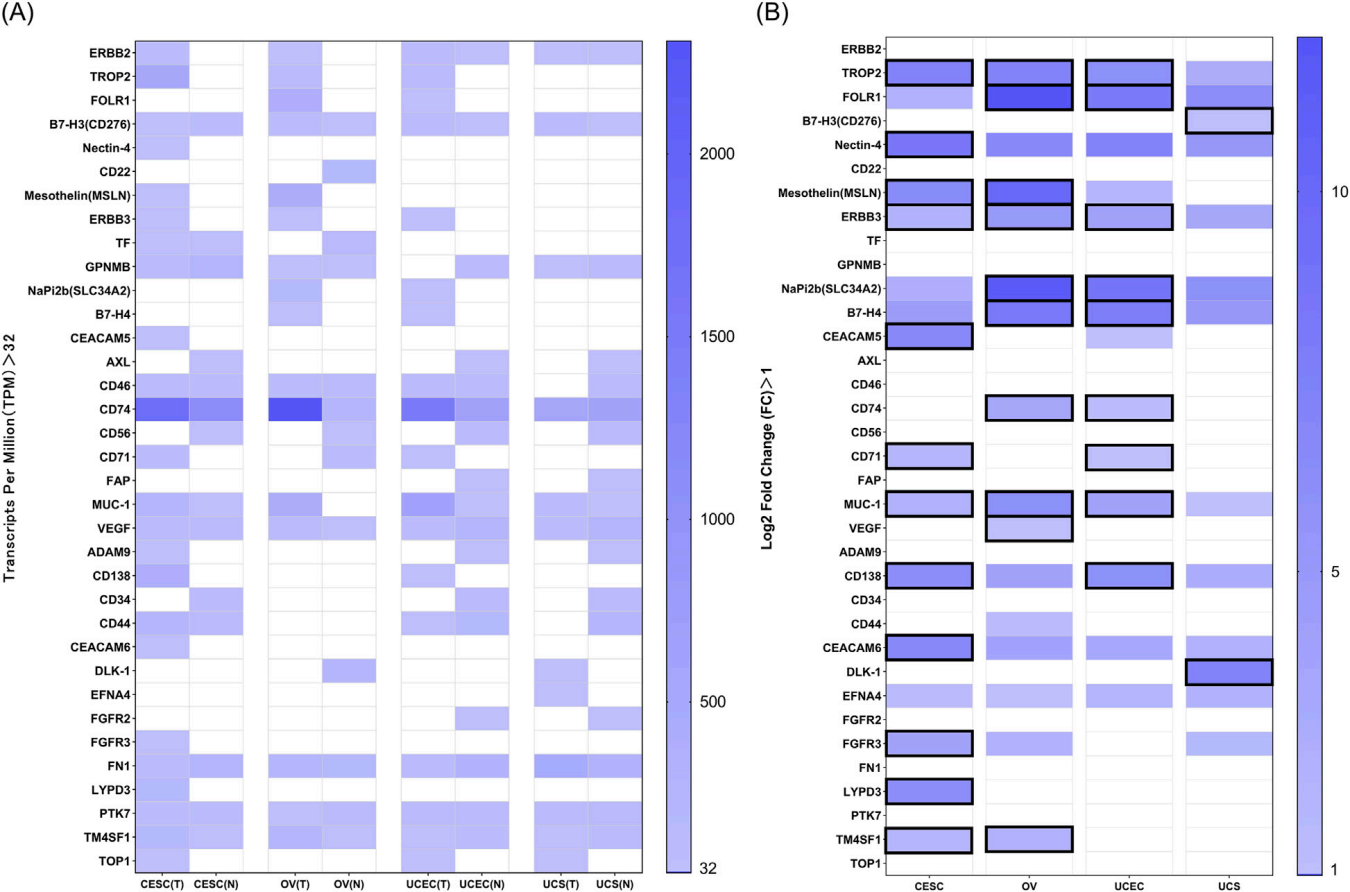
**(A)** RNA expression in TPM according to GEPIA2 of targets in normal and tumor tissues of four common gynecological malignancies. A value of 32 TPM is considered a medium-high expression. **(B)** Log2 Fold Change (Log2 FC) data between normal vs. tumor tissue for the above genes in the four tumors. The Log2 FC has been calculated in such a way that a value of 0 means equal expression, and a value of one means double expression in tumor tissue. Log2 FC greater than 1, and with expression more than 32 TPM are marked in black solid boxes.

We utilized gene expression data provided by GEPIA2 to evaluate the expression of these TATs in normal and tumor tissues of four common gynecological malignancies (Supplementary Figure 1, Supplementary File 3). We set the TPM value greater than 32, as this threshold is often used to define medium or high levels of gene expression. We screened for genes that exhibited medium or high expression in these four malignancies (Figure 2A). We defined the fold change as the ratio of tumor tissue TPM to normal tissue TPM (TPM tumor tissue/TPM normal tissue). We set Log2 Fold Change greater than 1, indicating that the expression in tumor tissue is more than twice that of normal tissue, suggesting that the gene is more significantly expressed in tumor tissue (Figure 2B). We marked the genes that meet both the “TPM greater than 32” and the “Log2 Fold Change greater than 1” with a solid black box. These are some TATs that are overexpressed in four types of gynecological tumors.

Among these TATs, we found some targets of FDA-approved ADCs that are widely expressed in unapproved indications, such as gynecological cancers. For example, Enfortumab Vedotin is an ADC targeting Nectin-4, which is currently only approved for treating advanced urothelial carcinoma (Powles et al., 2024). However, we found that Nectin-4 is also widely expressed in cervical cancer; Sacituzumab Govitecan is the world’s first and only approved ADC drug targeting Trop-2 for the treatment of unresectable locally advanced or metastatic triple-negative breast cancer (Wahby et al., 2021), and the target Trop-2 is also widely expressed in endometrial cancer, ovarian cancer, and cervical cancer; Trastuzumab Deruxtecan also has shown promising activity in various types of late stage solid tumors with ERBB2 expression, including traditionally difficult to treat malignant tumors (AACR, 2023). The drug is currently being evaluated in clinical trials for its efficacy in endometrial cancer, particularly in ERBB2-positive patients. In addition, ERBB2 is widely expressed in ovarian cancer, cervical cancer, and uterine sarcoma. This provides us with some new clinical indications for ADCs.

It is worth noting that compared with normal tissues, TROP2, ERBB3, and MUC1 are significantly upregulated in cervical cancer, ovarian cancer, and endometrial cancer, while MSLN and TM4SF1 are significantly upregulated in cervical cancer and ovarian cancer; the expression of NaPi2b, B7-H4, and CD74 is significantly upregulated in ovarian cancer and endometrial cancer. TATs highly expressed in cervical cancer include CEACAM5, CD71, CD138, FGFR3, LYPD3, CEACAM6, etc.; VEGF is also highly expressed in ovarian cancer; CD71 and CD138 are highly expressed in endometrial cancer; the TATs highly expressed in uterine sarcoma include B7-H3, DLK1, and EFNA4. This provides a new approach for target selection of ADCs in the field of gynecological malignancies treatment in the future.

### 3.3 Target prediction of different pathological types of gynecological malignancies

#### 3.3.1 Target prediction of various pathological types of ovarian cancer

**FIGURE 3:**
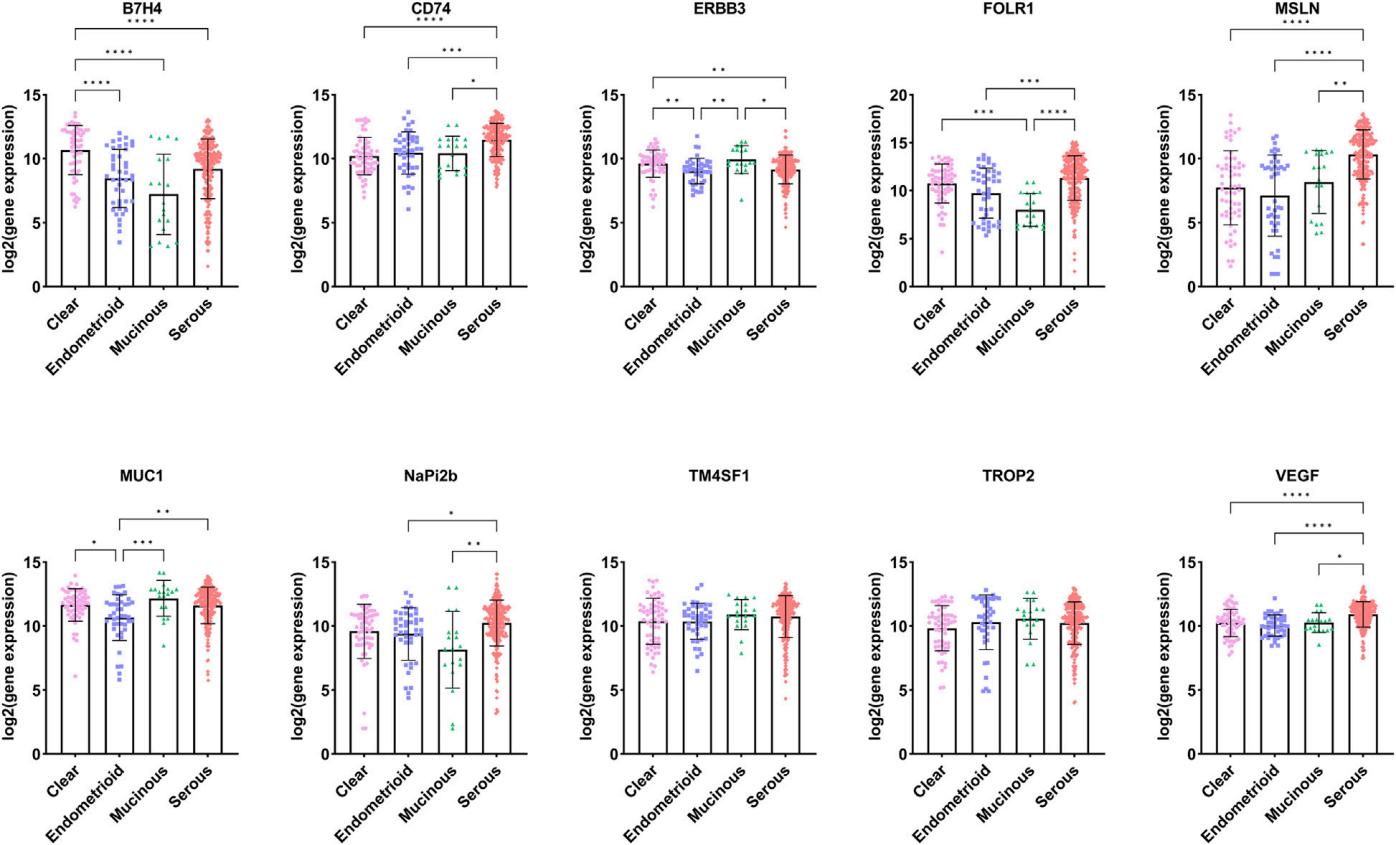
Expression of targets in different pathological types of ovarian cancer (Data sourced from GENT2. Clear: ovarian clear cell carcinoma; Endometrioid: ovarian endometrioid carcinoma; Mucinous: Mucinous ovarian cancer; Serous: Serous ovarian carcinoma).

Although FOLR1, NaPi2b, TROP2, and others are highly expressed in ovarian cancer, there are differences among different pathological types of epithelial ovarian cancer. We utilized the significantly upregulated TATs in ovarian cancer obtained in the previous section to further analyze the expression of these targets in different pathological types of ovarian cancer in the GENT2 database (Figure 3, Supplementary File 4). We selected four common pathological subtypes of ovarian cancer for discussion, including serous, mucinous, endometrioid and clear cell carcinoma. We observed that the expression of ERBB3 and MUC1 is higher in mucinous and clear than in serous and endometrioid carcinoma; the expression of B7H4 is higher in clear and serous than in mucinous and endometrioid; FOLR1 expression is significantly higher in serous, clear and endometrioid than in mucinous. In addition, target genes such as CD74, MSLN, NaPi2b and VEGF are more highly expressed in serous than in mucinous, clear and endometrioid carcinomas. There was no difference in the expression of TROP2 and TM4SF1 between the four ovarian cancer subtypes. Through in-depth analysis of the differential expression of specific molecular targets in different pathological types of ovarian cancer, it may help to provide more accurate treatment plans for ovarian cancer patients with different pathological types. In addition, this study paves the way for identifying new therapeutic targets and developing innovative treatment strategies, which is expected to significantly improve the effectiveness of ovarian cancer treatment and the quality of life of patients.

#### 3.3.2 Target prediction of various pathological types of endometrial cancer

**FIGURE 4:**
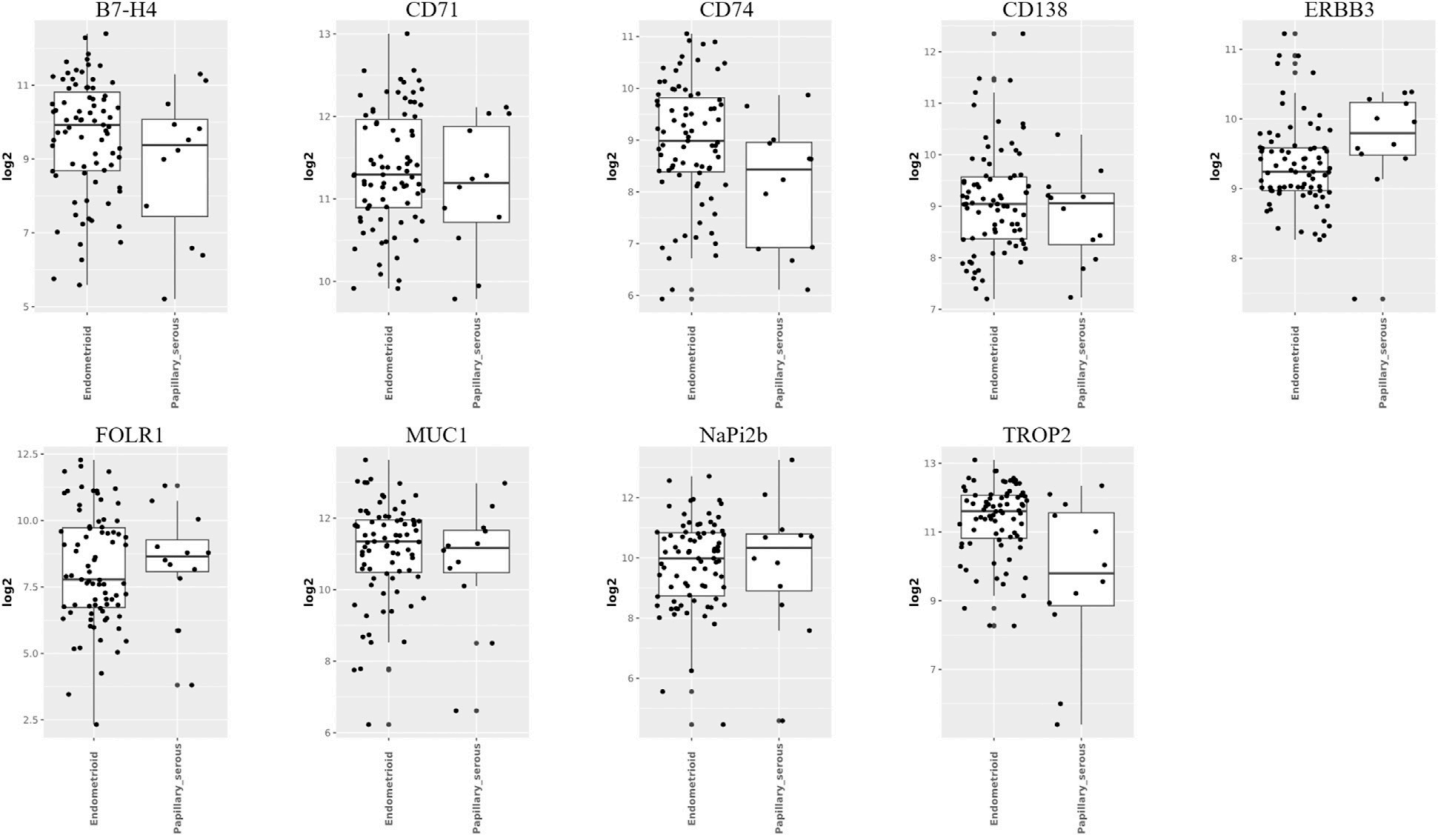
Expression of targets in different pathological types of endometrial cancer (Image sourced from GENT2. Endometrioid: endometrial endometrioid carcinoma; Papillary_serous: uterine serous carcinoma).

According to the WHO (2020) classification of female reproductive system tumors, endometrial cancer can be divided into endometrial endometrioid carcinoma (EEC), serous carcinoma (SC), clear cell carcinoma (CCC), mixed carcinoma (MC), etc. (Berek et al., 2023) Among them, the two most common pathological types of endometrial cancer are endometrioid carcinoma and serous carcinoma. We analyzed the TATs, which were significantly upregulated in endometrial cancer, through the GENT2 database (Figure 4). We found that among these targets, B7-H4, TROP2 and CD74 are upregulated considerably in endometrioid compared to serous cancer; there is no significant difference in the expression of CD71, MUC1, and CD138 between endometrioid and serous; the expression of ERBB3, FOLR1, and NaPi2b is higher in serous than in endometrioid carcinoma. Analyzing the differential expression of molecular targets in different pathological types of endometrial cancer is crucial for developing personalized and precise treatment strategies for patients.

#### 3.3.3 Target prediction for various pathological types of cervical cancer

Cervical cancer is the fourth most common malignant tumor that threatens women’s health worldwide. Currently, surgery, radiotherapy, and chemotherapy are still the main treatment options for cervical cancer patients. However, the treatment effect for advanced and recurrent metastatic cervical cancer patients is not ideal, which is also the main reason for the death of cervical cancer patients (Wang and Hao, 2024). We were committed to using the significant achievements of immunotherapy and targeted therapy in clinical trials to usher in a new era of treatment for advanced and recurrent metastatic cervical cancer. In this area, the birth of ADCs has attracted particular attention from the industry. At present, only one ADC approved by the FDA for use in cervical cancer is TV. We aim to conduct in-depth research on potential high expression targets in various pathological types of cervical cancer, in order to provide direction for the development of novel ADCs.

**FIGURE 5:**
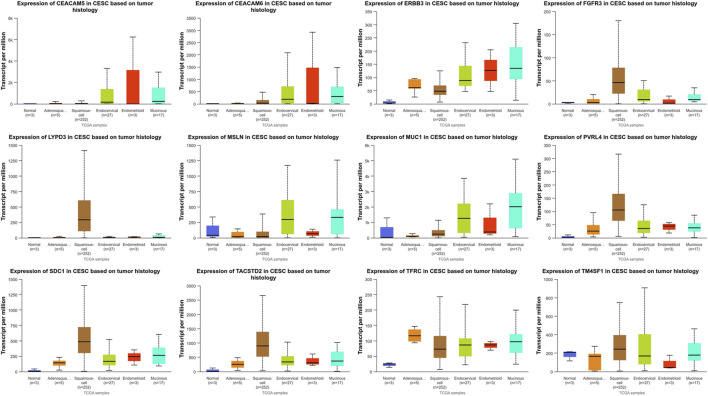
Expression of targets in different pathological types of cervical cancer (UALCAN) (Adenosquamous: Cervical adenosquamous carcinoma; Squamous-cell: Cervical squamous cell carcinoma; Endocervical: Endocervical adenocarcinoma of the usual type | Endocervical type of adenocarcinoma; Endometrioid: Endometrioid adenocarcinoma of endocervix; Mucinous: Mucinous adenocarcinoma of endocervical type. Definition: PVRL4, also known as Nectin-4; SDC1, alternatively denoted as CD138; TACSTD2, commonly referred to as TROP2; TFRC, also recognized as CD71).

Due to the lack of gene expression data for cervical tissue in the GENT2 database, we searched the UALCAN database for gene expression levels in cervical squamous cell carcinoma and cervical adenocarcinoma (CESC). In the UALCAN database, cervical cancer is classified into five histological subtypes: Adenosquamous cell carcinoma, Squamous-cell carcinoma, Endocervical adenocarcinoma, Endometrioid adenocarcinoma, Mucinous adenocarcinoma. The UALCAN database was employed to analyze the TATs previously identified as significantly upregulated in cervical cancer (Figure 5). Additionally, box plots of gene expression were generated for these genes. Our findings revealed that CEACAM5 and CEACAM6 exhibited comparable expression patterns across the five histological subtypes, with no notable statistical discrepancies. Conversely, the expression of ERBB3, MSLN, and MUC-1 was markedly elevated in adenocarcinoma relative to squamous cell carcinoma. FGFR3, LYPD3, PVRL4, SDC1, and TACSTD2 exhibited significantly elevated expression levels in squamous cell carcinoma relative to adenocarcinoma. Conversely, TFRC demonstrated higher expression in adenosquamous compared to squamous cell carcinoma and adenocarcinoma types. It is noteworthy that TM4SF1 exhibited significantly reduced expression in endometrioid adenocarcinoma compared to normal tissues.

### 3.4 Expression of common essential genes in gynecological malignancies

**FIGURE 6:**
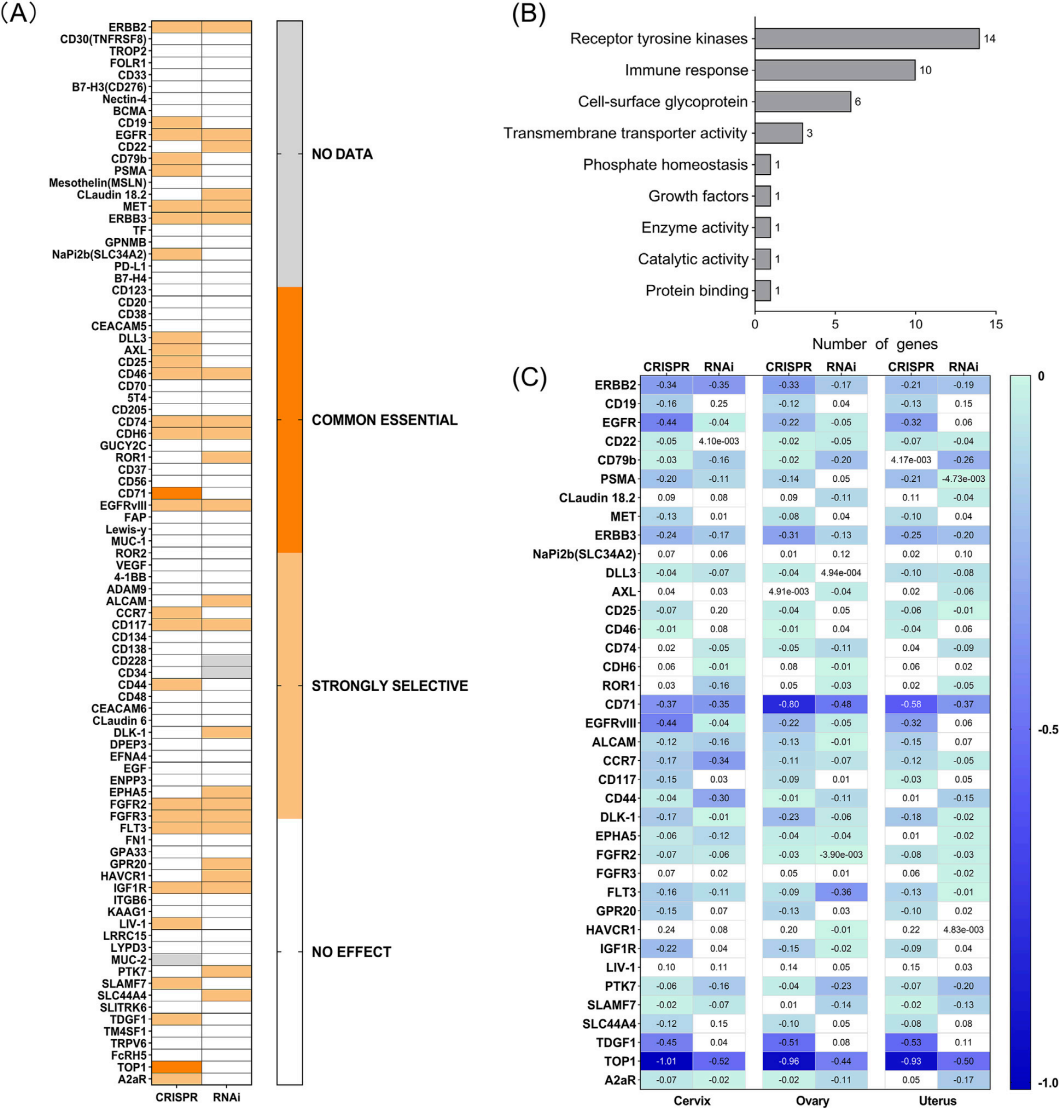
**(A)** Effect of silencing by both CRISPR and RNAi on different cell lines from several tumor types (DepMap). The scores appraise the effect size of knocking down or knocking out human genes. A negative score indicates that the cell lines grow slower after knocking down or knocking out a gene, while a positive score indicates that the cell lines grow faster. Common essential: A gene which, in a large, pan-cancer screen, ranks in the top most depleting genes in at least 90% of cell lines (the score of these genes is used as the dependent distribution for inferring dependency probability). Strongly selective: a gene whose dependency is at least 100 times more likely to have been sampled from a skewed distribution than a normal distribution. **(B)** Common essential and strongly selective gene function description. **(C)** Chronos and DEMETER2 dependency score of each gene in cervix, ovary, and uterus cancer (DepMap).

Above, we have identified TATs in tumor cells, but their relationship with tumor progression is still unclear. As described in the Materials and Methods section, we employed the comprehensive genome effects of CRISPR-Cas9 and RNAi provided by the DepMap database to identify essential and highly selective genes in tumor cells (Supplementary File 5). Among these TATs, two essential genes and several strongly selective genes were identified (Figure 6A). Essential genes include CD71 and TOP1, which play a central role in maintaining tumor cell survival and proliferation. This also implies that their downregulation can affect tumor cell proliferation; Strong selective genes include ERBB2, CD19, EGFR, CD22, CD79b, PSMA, Claudin-18.2, MET, ERBB3, NaPi2b, DLL3, AXL, CD25, CD46, CD74, CDH6, ROR1, ALCAM, CCR7, CD117, CD44, DLK-1, EPHA5, FGFR2, FGFR3, FLT3, GPR20, HAVCR1, IGF1R, LIV-1, PTK7, SLAMF7, SLC44A4, TDGF1 and A2aR, they exhibit significant dependence in specific cellular contexts. The subsequent step was to ascertain whether these targets played a role in carcinogenesis. The anti-tumor effect of ADCs can be generated not only through the action of the payload, but also through the inactivation of the carcinogenic effect of membrane receptors (Nieto-Jiménez et al., 2023). By accessing the gene function description page on the DepMap website, it was observed that the functions of these genes are mainly related to the signaling and immune response of receptor tyrosine kinases (RTKs) (Figure 6B). Our findings indicate the potential involvement of these genes in regulating tumor cell signaling and immune surveillance. Consequently, developing ADCs targeting these essential genes may prove more effective in exerting anti-tumor effects.

Furthermore, the decrease in expression values observed following gene knockout or knockdown in DepMap represents a significant phenotypic indicator, facilitating the elucidation of gene functions in tumor cells and their potential roles in cancer development. The perturbation effects indicate the impact of gene knockout on cell survival (Tsherniak et al., 2017). The more negative the gene effect value, the greater the impact on cell survival/proliferation after gene knockout. The closer the gene effect is to −1, the stronger the dependence of the cell line on this gene. A value greater than 0 indicates that this gene is not an essential gene for the cell line. By analyzing the dependency scores of Chronos and DEMETER2 for these genes in gynecological tumor cell lines (Figure 6C), we selected a threshold of −0.4 to screen for genes that inhibit more closely to the necessary level. We observed that CD71, TDGF1, and TOP1 are some of the most critical targets in cervical, ovarian, and uterine tumors, closely related to the survival/proliferation of cancer cells; ERBB2, EGFR, EGFR VIII are closely associated with the survival/proliferation of cervical cancer cells; other common basic targets include FLT3 in ovarian cancer. The above data provides a better understanding of the relationship between these genes and gynecological tumor cells. By downregulating their expression, they may have anti-cancer effects and offer clues to developing new ADCs for targeted treatment of gynecological cancers.

## 4 Discussion

### 4.1 Potential advantages and challenges of ADCs in gynecological malignancies

The emergence of ADCs marks the beginning of a new era in treating gynecological malignancies. While small molecule toxins exhibit potent cytotoxicity, the risk of direct administration is too high. Therefore, the design principle of ADCs is to utilize the ability of antibodies to bind to specific antigens on the surface of tumor cells, deliver toxins into tumor cells, and exert their cytotoxic effects to combat tumors (Zhang et al., 2020). The innovative combination of the large molecule targeting properties of antibodies and the killing effect of small molecule toxins in this type of drug provides a new treatment option for gynecological malignancy patients. With a deeper understanding of the biology of gynecological malignancies, especially for the three most common gynecological cancers of cervical cancer, endometrial cancer, and ovarian cancer, the necessity of ADCs is becoming increasingly prominent (Xia et al., 2023). The objective of this study was to evaluate the clinical development of current ADCs in gynecological malignancies. By retrieving data from ongoing clinical studies, our aim is to gain a rapid understanding of the current development of ADCs, with a view to enhancing their clinical application in gynecological cancers.

Despite the significant technological advantages and clinical application prospects of ADCs, the specificity of antibodies, the selection of target antigens, the type of toxins, and the linker or conjugation techniques are pivotal factors that determine the success or failure of ADC development.

Firstly, it is evident that most ADCs currently in development are based on traditional monoclonal antibody structures, with only a few containing selective inhibitors or bispecific antibodies. This is mainly because these novel ADCs have certain limitations in production and manufacturing. This may include high production costs, complex production processes (Walsh et al., 2021), and severe adverse reactions (Pegram et al., 2021). Due to these limitations, the clinical application progress of these novel ADCs is slow, and it is necessary to gradually overcome production challenges to promote them in clinical practice gradually (Gu et al., 2024).

Secondly, when analyzing the overexpression of TATs in gynecological malignancies, we observed that most of them are well-known targets, such as TROP2, FOLR1, MSLN, Nectin-4, etc. This not only highlights the bottleneck faced by ADCs in selecting new targets, but also reveals opportunities to explore other cell surface highly expressed proteins as potential targets.

In addition, there are two representative payloads of ADCs: microtubule inhibitors and DNA inhibitors, which are further divided into DNA-damaging agents and topoisomerase inhibitors. Most ADCs approved by the FDA use toxins such as sea hare toxins (MMAE, MMAF) and metformin (DM1, DM4), which are microtubule inhibitors. It is worth noting that toxicity is a crucial challenge factor limiting the therapeutic window of ADCs(Zhang et al., 2020), and many ADCs have failed in clinical development due to excessive toxicity. The main manifestations of toxicity largely depend on the type of payload (Zahavi and Weiner, 2020). Clinical data analysis shows that the vast majority of dose-limiting toxicity (DLT) is not caused by ADC drugs targeting normal tissues (Saber and Leighton, 2015). Different ADCs, even if targeting different antigens, often have similar dose-limiting toxicity if carrying the same payload (Nguyen et al., 2023). Microtubule inhibitors induce cell apoptosis by inhibiting microtubule polymerization, and DLTs with such payloads typically exhibit bone marrow suppression, such as neutropenia and thrombocytopenia, as well as peripheral neuropathy. MMAE reported G3/4 anemia, neutropenia, and peripheral neuropathy, DM1 reported thrombocytopenia and hepatotoxicity, and MMAF reported ocular toxicity (Masters et al., 2018). Among DNA inhibitors, DNA damaging agents such as kanamycin and PBD, as well as topoisomerase inhibitors such as camptothecin derivatives, may cause different toxic reactions, such as liver toxicity or gastrointestinal reactions (Nguyen et al., 2023).

Finally, linkers play a crucial role in the effectiveness of ADCs. Although non cleavable linkers exhibit higher stability in plasma, ADCs drugs also have better tolerance *in vivo*. However, in the majority of ADCs approved by the FDA, particularly those utilized in solid tumors, cleavable linkers are the preferred option. This is due to the fact that cleavable molecules acting as linkers can influence the activation of specific sites and the killing effect of bystanders, which is of particular importance for solid tumors that typically express multiple antigens (Kondrashov et al., 2023). The cleavable linker is crucial for inducing bystander effect (Bargh et al., 2019). ADCs containing cleavable linkers are internalized by tumor cells with high antigen expression, and the drug is degraded in lysosomes, releasing free toxins that can directly kill target cells or reach the tumor microenvironment, attacking surrounding tumor cells with low or no antigen expression (known as bystander cells), thereby exhibiting the bystander effect (Giugliano et al., 2022). However, the bystander killing effect may also pose some challenges, such as increasing the risk of non-specific toxicity, as the payload may affect non tumor tissues. Therefore, the design of ADC needs to find a balance between achieving bystander killing effect and controlling non-specific toxicity.

### 4.2 New targeting opportunities for ADCs in the treatment of gynecological malignancies

At present, ADCs have shown significant anti-tumor effects in monotherapy for various solid tumors and hematological malignancies and are considered a transformative treatment method. Despite the advances made by ADCs in the treatment of other cancers, their application in gynecological cancers remain limited. Currently, only Tisotumab Vedotin (TV) and Mirvetuximab Soravtansine (MIRV) have received FDA approval. Although these two drugs have demonstrated promise in the treatment of gynecological malignancies, further research and development of new ADCs are required to enhance treatment efficacy and meet the treatment needs of a larger patient population.

Until now, no scholars have conducted comprehensive assessments of the expression of targets and the selection of targets for ADCs in gynecological malignancies. Therefore, in this article, we evaluated the current clinical development process of ADCs, with a specific emphasis on targets that are highly expressed in gynecological malignancies. Our study aims to identify more targeted and therapeutic potential ADC targets, thereby providing a scientific foundation for future ADC development.

We used TPM and Fold Change to measure the expression levels of target genes of ADCs collected from current clinical trials and evaluated the expression of TATs in four common gynecological tumors through genomic data. We have identified TATs of some FDA-approved ADCs that are widely expressed in unapproved indications, which has provided us with new clinical indications for ADCs. For example, Enfortumab Vedotin and Sacituzumab Govitecan, although currently only approved for specific types of cancer treatment, their targets have also shown high expression in other gynecological malignancies, providing possibilities for exploring new indications for these drugs. We also discovered some TATs that are overexpressed in gynecological malignancies but have not yet been approved, which may provide new directions for developing ADCs. These findings emphasize the necessity of further clinical research on these potential targets to validate their effectiveness and safety in the treatment of gynecological tumors. We hope to provide patients with more treatment options and improve treatment outcomes through these studies.

In addition, different pathological types of ovarian cancer, endometrial cancer, and cervical cancer may have different target expression patterns. We use the GENT2 and UALCAN databases to predict and analyze potential therapeutic targets for different pathological types of gynecological malignancies by identifying significantly upregulated TATs in gynecological malignancies. In the ovarian cancer study, we observed that the expression levels of ERBB3 and MUC1 were higher in mucinous and clear cell carcinoma than in serous and endometrioid carcinoma. The expression level of B7H4 is higher in clear cell carcinoma and serous carcinoma than in mucinous and endometrioid carcinoma. The expression level of FOLR1 is significantly higher in serous, clear cell and endometrioid carcinoma than in mucinous carcinoma. In addition, the expression levels of target genes CD74, MSLN, NaPi2b, and VEGF are higher in serous carcinoma than in mucinous, clear cell and endometrioid carcinoma. However, there was no significant difference in the expression of TROP2 and TMFSF1 among the four histological subtypes of ovarian cancer. The two most common pathological types in endometrial cancer are endometrioid carcinoma and serous carcinoma. B7-H4, TROP2, and CD74 expression levels are significantly higher in endometrioid carcinoma than in serous carcinoma. There is no significant difference in the expression of CD71, MUC1, and CD138 between these two types of cancer. In contrast, ERBB3, FOLR1, and NaPi2b expression levels are higher in serous carcinoma than in endometrioid carcinoma. In the study of cervical cancer, the expression of CEACAM5 and CEACAM6 was similar in five different histological subtypes, with no significant statistical difference. ERBB3, MSLN, and MUC-1 expression was markedly elevated in adenocarcinoma relative to squamous cell carcinoma. FGFR3, LYPD3, PVRL4, SDC1, and TACSTD2 exhibited significantly elevated expression levels in squamous cell carcinoma relative to adenocarcinoma. TFRC demonstrated higher expression in adenosquamous compared to squamous cell carcinoma and adenocarcinoma types. Of particular note is that the expression of TM4SF1 in endometrioid adenocarcinoma is significantly lower than in normal tissues.

Due to the unclear relationship between these TATs and tumor progression. To investigate whether these TATs are involved in the carcinogenic process or play a carcinogenic role, we conducted an analysis using the DepMap database. It is worth noting that we have found that specific genes, such as CD71 and TOP1, play an indispensable role in gynecological tumor cell lines and may be key factors driving cancer development. In addition, there are strong selective genes such as TDGF1, EGFR, and FLT3, whose downregulation can also inhibit tumor growth. Our research reveals the link between these genes and gynecological tumor cells, indicating that regulating their expression levels may have inhibitory effects on tumor cells. These findings not only enhance our understanding of the role of these genes in gynecological tumors, but also provide valuable clues for the development of targeted antibody-drug conjugates. It is worth noting that in the previous analysis of the expression of TATs in normal and tumor tissues of four common gynecological tumors, we found that CD71 (also known as transferrin receptor) is a target overexpressed on the surface of gynecological malignant tumor cells. In addition, CD71 is an essential gene for tumor cells, and downregulating its expression can inhibit tumor growth. Therefore, we infer that CD71 is a potential target for antibody-drug conjugates. However, as CD71 belongs to the transferrin receptor and is widely expressed in normal cells, it has traditionally been challenging to develop ADCs targeting this target. However, CX-2029 overcomes this problem through the innovative design of its precursor (Johnson et al., 2021). We also hope that novel ADCs targeting CD71 may provide new treatment options for patients with difficult to treat gynecological malignancies in the future.

## 5 Conclusion

Although ADCs have made some progress in the treatment of gynecological malignancies, the current range of approved drugs remains limited. Consequently, further research and development of more ADCs are needed for selection. However, the choice of targets in targeted therapy for gynecological malignancies has not been thoroughly evaluated. This article discusses the current clinical progress of ADCs, focusing on targets highly expressed in gynecological malignancies. It deeply analyzes the differential expression of TATs in different pathological types of gynecological malignant tumors, as well as targets closely related to the survival and proliferation of cancer cells. This provides a scientific foundation for the selection of novel targets for developing ADCs. The main limitation of this study is that it was conducted at the transcriptome level, with no inclusion of protein data. Additionally, since our assessment of gene expression was based on public databases, they did not provide the sample size for each analysis (tumor or normal). Moreover, information about the characteristics of the samples, the stage of the disease, prognostic factors, previous treatments, etc., are unknown. These risk factors could affect the results. Furthermore, the development of improved ADC designs that enhance stability and targeting while reducing toxicity to normal cells represents a current research priority. In conclusion, through the continuous optimization of design and the exploration of clinical applications, we hope that more ADCs can bring more effective and precise treatment to patients with gynecological malignancy.

## Data Availability

The original contributions presented in the study are included in the article/Supplementary Material, further inquiries can be directed to the corresponding author.

## References

[B1] AACR (2023). HER2-Targeted therapy shows promise across tumor types. Cancer Discov. 13 (8), 3. 10.1158/2159-8290.Cd-nb2023-0047 37279357

[B2] Bargh J. D. Isidro-Llobet A. Parker J. S. Spring D. R. (2019). Cleavable linkers in antibody-drug conjugates. Chem. Soc. Rev. 48 (16), 4361–4374. 10.1039/c8cs00676h 31294429

[B3] Berek J. S. Matias-Guiu X. Creutzberg C. Fotopoulou C. Gaffney D. Kehoe S. (2023). FIGO staging of endometrial cancer: 2023. Int. J. Gynaecol. Obstet. 162 (2), 383–394. 10.1002/ijgo.14923 37337978

[B4] Boghaert E. R. Cox M. C. Vaidya K. S. (2022). Pathophysiologic and pharmacologic considerations to improve the design and application of antibody-drug conjugates. Cancer Res. 82 (10), 1858–1869. 10.1158/0008-5472.Can-21-3236 35298624

[B5] Chari R. V. (2008). Targeted cancer therapy: conferring specificity to cytotoxic drugs. Acc. Chem. Res. 41 (1), 98–107. 10.1021/ar700108g 17705444

[B6] Díaz-Rodríguez E. Gandullo-Sánchez L. Ocaña A. Pandiella A. (2021). Novel ADCs and strategies to overcome resistance to anti-HER2 ADCs. Cancers (Basel) 14 (1), 154. 10.3390/cancers14010154 35008318 PMC8750930

[B7] Drago J. Z. Modi S. Chandarlapaty S. (2021). Unlocking the potential of antibody-drug conjugates for cancer therapy. Nat. Rev. Clin. Oncol. 18 (6), 327–344. 10.1038/s41571-021-00470-8 33558752 PMC8287784

[B8] Dumontet C. Reichert J. M. Senter P. D. Lambert J. M. Beck A. (2023). Antibody-drug conjugates come of age in oncology. Nat. Rev. Drug Discov. 22 (8), 641–661. 10.1038/s41573-023-00709-2 37308581

[B9] Ford C. H. Newman C. E. Johnson J. R. Woodhouse C. S. Reeder T. A. Rowland G. F. (1983). Localisation and toxicity study of a vindesine-anti-CEA conjugate in patients with advanced cancer. Br. J. Cancer 47 (1), 35–42. 10.1038/bjc.1983.4 6821632 PMC2011264

[B10] Fu C. Tong W. Yu L. Miao Y. Wei Q. Yu Z. (2024). When will the immune-stimulating antibody conjugates (ISACs) be transferred from bench to bedside? Pharmacol. Res. 203, 107160. 10.1016/j.phrs.2024.107160 38547937

[B11] Giugliano F. Corti C. Tarantino P. Michelini F. Curigliano G. (2022). Bystander effect of antibody-drug conjugates: fact or fiction? Curr. Oncol. Rep. 24 (7), 809–817. 10.1007/s11912-022-01266-4 35305211

[B12] Gu Y. Wang Z. Wang Y. (2024). Bispecific antibody drug conjugates: making 1+1>2. Acta Pharm. Sin. B 14 (5), 1965–1986. 10.1016/j.apsb.2024.01.009 38799638 PMC11119582

[B13] Heitz N. Greer S. C. Halford Z. (2023). A review of Tisotumab vedotin-tftv in recurrent or metastatic cervical cancer. Ann. Pharmacother. 57 (5), 585–596. 10.1177/10600280221118370 35962528

[B14] Johnson M. El-Khoueiry A. Hafez N. Lakhani N. Mamdani H. Rodon J. (2021). Phase I, first-in-human study of the probody therapeutic CX-2029 in adults with advanced solid tumor malignancies. Clin. Cancer Res. 27 (16), 4521–4530. 10.1158/1078-0432.Ccr-21-0194 34083236

[B15] Kondrashov A. Sapkota S. Sharma A. Riano I. Kurzrock R. Adashek J. J. (2023). Antibody-drug conjugates in solid tumor oncology: an effectiveness payday with a targeted payload. Pharmaceutics 15 (8), 2160. 10.3390/pharmaceutics15082160 37631374 PMC10459723

[B16] Li Y. Zhang L. Li R. Zhang M. Li Y. Wang H. (2019). Systematic identification and validation of the reference genes from 60 RNA-Seq libraries in the scallop Mizuhopecten yessoensis. BMC Genomics 20 (1), 288. 10.1186/s12864-019-5661-x 30975074 PMC6460854

[B17] Masters J. C. Nickens D. J. Xuan D. Shazer R. L. Amantea M. (2018). Clinical toxicity of antibody drug conjugates: a meta-analysis of payloads. Invest New Drugs 36 (1), 121–135. 10.1007/s10637-017-0520-6 29027591

[B18] Meric-Bernstam F. Makker V. Oaknin A. Oh D. Y. Banerjee S. González-Martín A. (2024). Efficacy and safety of Trastuzumab deruxtecan in patients with HER2-expressing solid tumors: primary results from the DESTINY-PanTumor02 phase II trial. J. Clin. Oncol. 42 (1), 47–58. 10.1200/jco.23.02005 37870536 PMC10730032

[B19] Moore K. N. Angelergues A. Konecny G. E. García Y. Banerjee S. Lorusso D. (2023). Mirvetuximab soravtansine in frα-positive, platinum-resistant ovarian cancer. N. Engl. J. Med. 389 (23), 2162–2174. 10.1056/NEJMoa2309169 38055253

[B20] Nguyen T. D. Bordeau B. M. Balthasar J. P. (2023). Mechanisms of ADC toxicity and strategies to increase ADC tolerability. Cancers (Basel) 15 (3), 713. 10.3390/cancers15030713 36765668 PMC9913659

[B21] Nieto-Jiménez C. Sanvicente A. Díaz-Tejeiro C. Moreno V. Lopez de Sá A. Calvo E. (2023). Uncovering therapeutic opportunities in the clinical development of antibody-drug conjugates. Clin. Transl. Med. 13 (9), e1329. 10.1002/ctm2.1329 37740463 PMC10517221

[B22] Park S. J. Yoon B. H. Kim S. K. Kim S. Y. (2019). GENT2: an updated gene expression database for normal and tumor tissues. BMC Med. Genomics 12 (Suppl. 5), 101. 10.1186/s12920-019-0514-7 31296229 PMC6624177

[B23] Pegram M. D. Hamilton E. P. Tan A. R. Storniolo A. M. Balic K. Rosenbaum A. I. (2021). First-in-Human, phase 1 dose-escalation study of biparatopic anti-HER2 antibody-drug conjugate MEDI4276 in patients with HER2-positive advanced breast or gastric cancer. Mol. Cancer Ther. 20 (8), 1442–1453. 10.1158/1535-7163.Mct-20-0014 34045233 PMC9398097

[B24] Phuna Z. X. Kumar P. A. Haroun E. Dutta D. Lim S. H. (2024). Antibody-drug conjugates: principles and opportunities. Life Sci. 347, 122676. 10.1016/j.lfs.2024.122676 38688384

[B25] Powles T. Valderrama B. P. Gupta S. Bedke J. Kikuchi E. Hoffman-Censits J. (2024). Enfortumab Vedotin and pembrolizumab in untreated advanced urothelial cancer. N. Engl. J. Med. 390 (10), 875–888. 10.1056/NEJMoa2312117 38446675

[B26] Riccardi F. Dal Bo M. Macor P. Toffoli G. (2023). A comprehensive overview on antibody-drug conjugates: from the conceptualization to cancer therapy. Front. Pharmacol. 14, 1274088. 10.3389/fphar.2023.1274088 37790810 PMC10544916

[B27] Saber H. Leighton J. K. (2015). An FDA oncology analysis of antibody-drug conjugates. Regul. Toxicol. Pharmacol. 71 (3), 444–452. 10.1016/j.yrtph.2015.01.014 25661711

[B28] Strebhardt K. Ullrich A. (2008). Paul Ehrlich's magic bullet concept: 100 years of progress. Nat. Rev. Cancer 8 (6), 473–480. 10.1038/nrc2394 18469827

[B29] Tsherniak A. Vazquez F. Montgomery P. G. Weir B. A. Kryukov G. Cowley G. S. (2017). Defining a cancer dependency map. Cell 170 (3), 564–576. 10.1016/j.cell.2017.06.010 28753430 PMC5667678

[B30] Veneziani A. C. Sneha S. Oza A. M. (2024). Antibody-drug conjugates: advancing from magic bullet to biological missile. Clin. Cancer Res. 30 (8), 1434–1437. 10.1158/1078-0432.Ccr-23-3414 38306232

[B31] Wahby S. Fashoyin-Aje L. Osgood C. L. Cheng J. Fiero M. H. Zhang L. (2021). FDA approval summary: accelerated approval of Sacituzumab govitecan-hziy for third-line treatment of metastatic triple-negative breast cancer. Clin. Cancer Res. 27 (7), 1850–1854. 10.1158/1078-0432.Ccr-20-3119 33168656

[B32] Walsh S. J. Bargh J. D. Dannheim F. M. Hanby A. R. Seki H. Counsell A. J. (2021). Site-selective modification strategies in antibody-drug conjugates. Chem. Soc. Rev. 50 (2), 1305–1353. 10.1039/d0cs00310g 33290462

[B33] Wang W. Hao M. (2024). Immunotherapy and targeted therapy for advanced or recurrent/metastatic cervical cancer. Chin. J. Clin. Obstetrics Gynecol. 25 (05), 385–387. 10.13390/j.issn.1672-1861.2024.05.001

[B34] Xia L. Zhu J. Wu X. (2023). The latest progress and prospect of gynecological tumor treatment at 2023 ESMO. China Oncol. 33 (11), 969–980. 10.19401/j.cnki.1007-3639.2023.11.001

[B35] Zahavi D. Weiner L. (2020). Monoclonal antibodies in cancer therapy. Antibodies (Basel) 9 (3), 34. 10.3390/antib9030034 32698317 PMC7551545

[B36] Zhang B. Zhou H. Lin Z. He S. li J. D. Yang L. (2024). Guidelines for the clinical application of antibody-drug conjugate for gynecological malignancies(2024 edition). Chineses J. Pract. Gynecol. Obstetrics 40 (05), 516–525. 10.19538/j.fk2024050111

[B37] Zhang Z. Wang Y. Bai Y. (2020). Consideration of antibody drug conjugates development and regulation. Consid. Antib. drug conjugates Dev. Regul. 55 (08), 1971–1977. 10.16438/j.0513-4870.2020-0325

